# Cardiovascular risk profile and treatment patterns in patients with type 2 diabetes in specialized cardiology outpatient care in Colombia: A retrospective cross-sectional study

**DOI:** 10.1097/MD.0000000000051307

**Published:** 2026-10-23

**Authors:** Lucia Suarez Maestre, Ingrith Paola Hoyos Montaño, Zenen David Rúa Osorio, Carlos Renowitzky Zabarain, Alex Dominguez Vargas, Henry J. González-Torres

**Affiliations:** a Centro de Investigaciones en Ciencias de la Vida, Facultad de Ciencias de la Salud, Universidad Simón Bolívar, Barranquilla, Colombia; b Departamento de Cardiología, Clínica Centro, Barranquilla, Colombia; c Data Analysis and Mining Department, Data and Project Consulting Service SAS, Barranquilla, Colombia.

**Keywords:** cardiovascular diseases, cardiovascular risk, Colombia, diabetes mellitus, type 2, echocardiography, glycated hemoglobin

## Abstract

Type 2 diabetes mellitus (T2DM) is strongly associated with cardiovascular morbidity, yet real-world evidence integrating cardiovascular comorbidities, metabolic characteristics, echocardiographic findings, and treatment patterns remains limited in Colombia. This study characterized the cardiovascular profile and treatment patterns of patients with T2DM attending specialized cardiology care and explored factors associated with glycated hemoglobin (HbA1c) >7%. A single-center, retrospective cross-sectional study included 138 adults with confirmed T2DM attending a high-complexity cardiology outpatient clinic in Barranquilla, Colombia, during 2023 to 2024. Patients were classified as HbA1c ≤7% or >7% using the most recent value documented at the latest outpatient visit. Clinical, laboratory, electrocardiographic, echocardiographic, and prescribed medication variables were evaluated. Univariable analyses were followed by an exploratory multivariable logistic regression model using the enter method. Mean age was 67.3 ± 11.5 years, 71.7% were male, and 70.3% had HbA1c >7%. Patients with HbA1c >7% were older (68.6 ± 11.6 vs 64.2 ± 10.9 years; *P* = .034) and had higher total cholesterol (203.5 ± 48.1 vs 181.7 ± 42.8 mg/dL; *P* = .010) and low-density lipoprotein cholesterol (124.9 ± 36.1 vs 106.0 ± 39.1 mg/dL; *P* = .010). Cerebrovascular disease occurred in 11.3% of patients with HbA1c >7% and none with HbA1c ≤7% (*P* = .034). The absence of prespecified echocardiographic abnormalities was less frequent with HbA1c >7% (15.5% vs 34.1%; *P* = .014). Sodium–glucose cotransporter-2 (SGLT2) inhibitor use was documented in 16.7% overall and was more frequent with HbA1c ≤7% (26.8% vs 12.4%; *P* = .037). In the exploratory multivariable model, total cholesterol was associated with HbA1c >7% (adjusted odds ratio [OR] per 10 mg/dL = 1.10; 95% confidence interval [CI], 1.01–1.20; *P* = .033), whereas the absence of prespecified echocardiographic abnormalities was inversely associated (adjusted OR = 0.36; 95% CI, 0.15–0.88; *P* = .026). SGLT2 inhibitor use was not significant after adjustment (adjusted OR = 0.56; 95% CI, 0.20–1.58; *P* = .274). In this Colombian real-world cohort, HbA1c >7% was common. Higher total cholesterol was positively associated with HbA1c >7%, whereas the absence of prespecified echocardiographic abnormalities was inversely associated with this outcome. The association with SGLT2 inhibitor use did not persist after adjustment. These cross-sectional findings support integrated cardiometabolic assessment in specialized outpatient care.

## 1. Introduction

Type 2 diabetes mellitus (T2DM) represents one of the major global public health challenges due to its high prevalence, chronic progression, and strong association with cardiovascular complications.^[1–3]^ While traditional paradigms focused primarily on atherosclerotic risk reduction, contemporary evidence highlights heart failure (HF) and structural myocardial disease as early, highly prevalent, yet profoundly underappreciated complications. In this context, glycemic control, dyslipidemia, and other modifiable risk factors play a crucial role in the progression of these complications.^[4,5]^

Currently, the clinical management of T2DM in cardiology faces significant controversies. There is a critical knowledge gap regarding the true burden of subclinical structural heart disease, such as left ventricular hypertrophy and early diastolic dysfunction, in real-world asymptomatic outpatients. This emphasizes the need for comprehensive laboratory and imaging profiling beyond routine metabolic assessments.^[6,7]^ In Colombia, recent population-based evidence has demonstrated a substantial burden of T2DM and associated cardiometabolic conditions. A representative cross-sectional survey conducted in Bogotá in 2022 to 2023 estimated a T2DM prevalence of 11.0% and a prevalence of metabolic syndrome ranging from 29.3% to 33.9%, depending on the diagnostic criteria used.^[8]^ However, this study was population-based and did not specifically characterize patients receiving specialized cardiovascular care.

Using longitudinal real-world data from 969,531 Colombian adults with diabetes, Urina-Jassir et al found that 85% had at least 1 major comorbidity. Chronic kidney disease and obesity were associated with higher odds of poor glycemic control (odds ratio [OR] 1.78 and 1.52, respectively), whereas hypertension was not independently associated with this outcome.^[9]^ Although this national registry provided important information on the relationship between comorbidity burden and glycemic control, it did not include an integrated assessment of electrocardiographic, echocardiographic, lipid, and treatment characteristics in specialized cardiology settings.

In the Colombian Caribbean, Miranda-Machado et al reported that T2DM was associated with a higher risk of a 1st cardiovascular event (hazard ratio [HR] 1.69), myocardial infarction (HR 1.79), and stroke (HR 1.54) in patients enrolled in a cardiovascular risk-management program.^[10]^ Nevertheless, that study focused on incident cardiovascular outcomes rather than the clinical, paraclinical, and therapeutic profile of patients with T2DM already receiving specialized outpatient cardiology care.

Furthermore, a major translational gap exists between current clinical guidelines and daily practice, often referred to as “therapeutic inertia.”^[11]^ On 1 hand, international guidelines recommend the early use of therapies with proven cardiovascular benefit, such as sodium–glucose cotransporter-2 (SGLT2) inhibitors and glucagon-like peptide-1 receptor agonists, even independently of glycemic control.^[12–14]^ On the other hand, studies have documented low utilization of these therapies, attributed to factors such as clinical inertia, economic barriers, and healthcare system limitations.^[15–17]^ This discrepancy is particularly pronounced in developing regions and among patients within subsidized healthcare systems, where socioeconomic barriers and fragmented care models severely hinder the adoption of disease-modifying drugs.^[18,19]^

Additionally, cardiovascular risk assessment in patients with T2DM presents methodological challenges. Widely used tools such as systematic coronary risk evaluation 2 and pooled cohort equations-American College of Cardiology/American Heart Association have shown limitations in their predictive performance in Latin American populations, often overestimating risk and requiring local recalibration.^[20]^ This uncertainty complicates clinical decision-making and may influence the selection of preventive and therapeutic strategies. In this context, the cardiology outpatient setting represents a key scenario for comprehensive cardiovascular risk assessment and treatment optimization in patients with T2DM. However, local evidence regarding the clinical, paraclinical, and therapeutic profile of these patients in specialized outpatient settings remains limited, particularly in regions such as the Colombian Caribbean.

Therefore, this study aimed to characterize the burden of clinical comorbidities, lipid abnormalities, subclinical structural heart disease, and treatment patterns among patients with T2DM in a specialized cardiology clinic in Barranquilla. By focusing on this underserved regional cohort, this work addresses a significant gap in Latin American evidence and identifies critical opportunities to optimize cardiometabolic management in daily clinical practice.

## 2. Materials and methods

### 2.1. Study design

An analytical, cross-sectional, retrospective study was conducted to evaluate the relationship between sociodemographic, clinical, paraclinical, and therapeutic variables and glycemic control in patients with T2DM. This study was designed, conducted, and reported in accordance with the Strengthening the Reporting of Observational Studies in Epidemiology guidelines for cross-sectional studies. The study aimed to identify patterns associated with cardiovascular conditions and treatment strategies in a specialized outpatient setting. The study was performed in a high-complexity cardiology outpatient clinic in Barranquilla, Colombia, and included medical records from patients attended during the 2023 to 2024 period.

### 2.2. Population and inclusion criteria

The study population comprised adult patients (≥18 years) with a confirmed diagnosis of T2DM who attended the specialized cardiology outpatient clinic during the 2023 to 2024 period. Eligible participants were required to have sufficient clinical information for the core variables needed for the primary analysis. Patients were strictly excluded if they had type 1 diabetes mellitus, secondary forms of diabetes, conditions that could significantly alter metabolic parameters at the time of evaluation (such as pregnancy or acute decompensated diseases), or incomplete clinical records for the main study variables. Consecutive sampling was employed, encompassing the eligible population evaluated during the study period. Of 845 medical records initially screened, 315 corresponded to patients with a confirmed diagnosis of T2DM. Among these, 177 were excluded because of incomplete records for the core variables required for the primary analysis, resulting in a final analytical cohort of 138 patients. The patient selection process is summarized in Figure 1.

**Figure 1.**
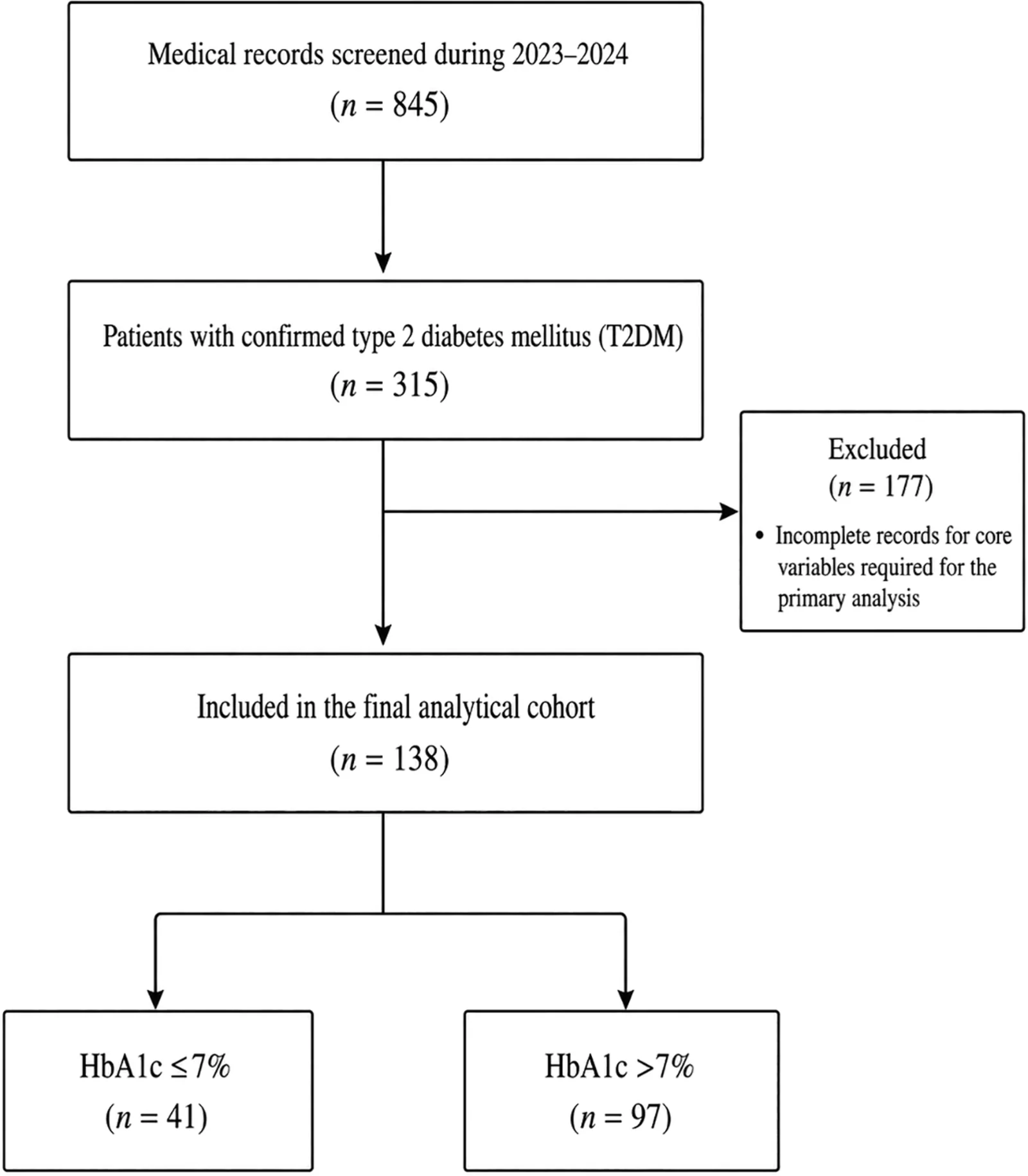
Flow chart of the study population selection. HbA1c = glycated hemoglobin.

### 2.3. Variables and operational definitions

The study included sociodemographic, clinical, electrocardiographic, paraclinical, therapeutic, and outcome variables obtained from electronic medical records. Sociodemographic variables included age, sex, and health insurance regime. Clinical variables comprised hypertension, ischemic heart disease (IHD), HF, atrial fibrillation (AF), cerebrovascular disease (CVD), and ventricular dysfunction (systolic or diastolic).

Electrocardiographic variables were obtained from formal 12-lead electrocardiogram (ECG) reports and included cardiac rhythm (sinus rhythm or AF), conduction abnormalities (1st-degree atrioventricular block, right bundle branch block, and left bundle branch block), ventricular extrasystoles, and electrocardiographic left ventricular hypertrophy.

Laboratory variables included glycated hemoglobin (HbA1c), a complete lipid profile (total cholesterol, low-density lipoprotein cholesterol, high-density lipoprotein cholesterol, and triglycerides), and renal function parameters (serum creatinine and estimated glomerular filtration rate, calculated using the modification of diet in renal disease [4-variable equation]). For each patient, the HbA1c value used for the analysis was the most recent measurement available in the medical record and documented at the patient’s latest outpatient cardiology visit. When multiple HbA1c measurements were available, only the most recent value was considered; measurements were not averaged.

Therapeutic variables included prescribed use of angiotensin-converting enzyme (ACE) inhibitors or angiotensin receptor blockers (ARB), beta-blockers, statins, antiplatelet agents, amiodarone, direct oral anticoagulants, and SGLT2 inhibitors. Medication use was ascertained from the medical prescription documented in the medical record at the patient’s most recent outpatient cardiology visit. Accordingly, these variables represent documented prescribed medication use rather than medication adherence, dispensing, or confirmed medication intake.

For analytical purposes, patients were categorized according to HbA1c ≤7% or >7%. The 7% threshold was used as a standardized population-level analytical cutoff and should not be interpreted as an individualized therapeutic target. Optimal HbA1c targets may differ according to age, comorbidities, functional status, hypoglycemia risk, and other patient-specific characteristics.^[21]^

### 2.4. Echocardiographic assessment

Transthoracic echocardiography (TTE) data were extracted from formal reports generated by board-certified cardiologists during routine high-complexity outpatient care. TTE parameters were acquired and interpreted in accordance with the American Society of Echocardiography and the European Association of Cardiovascular Imaging guidelines.^[22,23]^

Left ventricular ejection fraction (LVEF) was assessed primarily via the modified biplane Simpson’s method and categorized as ≥50%, 40% to 49%, or <40%. Systolic dysfunction was considered present when it was explicitly documented in the formal echocardiographic report, independently of the LVEF category. Diastolic dysfunction was determined from the formal echocardiographic interpretation based on guideline-recommended multiparametric assessment, including transmitral pulsed-wave Doppler and tissue Doppler parameters when available.^[22,23]^ Because the study was retrospective, individual echocardiographic measurements were not centrally re-adjudicated. Echocardiographic abnormalities were not considered mutually exclusive and could coexist in the same patient.

Because data were retrospectively extracted from routine clinical care, advanced imaging techniques such as speckle-tracking echocardiography were not systematically available across the cohort and were thus excluded from the analysis. For the present analysis, the previous “normal echocardiogram” variable was replaced by a composite variable termed “absence of prespecified echocardiographic abnormalities.” This variable was defined as an LVEF ≥ 50% together with the absence of hypokinesia, echocardiographic left ventricular hypertrophy, diastolic dysfunction, and systolic dysfunction. This composite was used for analytical purposes and should not be interpreted as evidence of completely normal cardiac structure or function.

### 2.5. Data sources and collection

Data were retrospectively collected from institutional electronic medical records. A standardized data extraction form was designed to ensure consistency in the collection of sociodemographic, clinical, paraclinical, and therapeutic variables. Each record was individually reviewed by the research team to verify data completeness and accuracy. The dataset was anonymized by assigning a unique numerical code to each patient and stored in a secure digital database prior to statistical analysis.

Complete information for the core variables required for the primary analysis, including HbA1c, lipid profile, and a formal echocardiographic report, was required for inclusion in the analytical cohort. Patients with incomplete records for these core variables were excluded during cohort selection. No statistical imputation methods were applied. For variables with missing observations within the final analytical cohort, descriptive and comparative analyses were based on the available observations for each variable, with denominators reflecting data availability.

Due to institutional and ethical restrictions, the dataset is not publicly available but can be made available from the corresponding author upon reasonable request.

### 2.6. Ethical considerations

The study protocol was reviewed and endorsed by the Scientific Committee of the Faculty of Health Sciences, Universidad Simón Bolívar (October 27, 2025), and subsequently approved by the Ethics Committee of Clínica Centro S.A. (protocol code 0001; Act CEICC-41; session No. 032; October 29, 2025), which classified the study as no-risk research. The requirement for informed consent was waived by the Ethics Committee because of the retrospective study design and the use of fully anonymized clinical data.

### 2.7. Statistical analysis

Descriptive statistics were used to summarize the data. Continuous variables were expressed as mean ± standard deviation after assessment of normality using the Shapiro–Wilk test. Categorical variables were presented as absolute frequencies and percentages. Comparisons between the HbA1c ≤7% and HbA1c >7% groups were performed using Welch’s *t* test for continuous variables and the Pearson chi-square test or Fisher’s exact test for categorical variables, as appropriate. Because HbA1c defined the analytical groups, its between-group comparison was considered descriptive, and no inferential *P* value was reported.

Univariable logistic regression analyses were performed with HbA1c >7% as the dependent outcome, and crude ORs with 95% confidence intervals (CIs) were estimated. In cases of complete separation, conventional logistic ORs were considered non-estimable, and Fisher’s exact test was used.

Given the sample size constraints, an exploratory multivariable logistic regression model was fitted using the enter method and included age, sex, total cholesterol, absence of prespecified echocardiographic abnormalities, and prescribed SGLT2 inhibitor use. No automated stepwise or *P* value-based selection procedure was used. Age and total cholesterol were modeled continuously per 10-year and 10-mg/dL increments, respectively; triglycerides were modeled per 50-mg/dL increment in univariable analyses. Cerebrovascular disease was excluded from the conventional multivariable model because the absence of events in the HbA1c ≤7% group resulted in complete or quasi-complete separation. IHD was additionally evaluated in a sensitivity model.

Multicollinearity was assessed using variance inflation factors, and the linearity-in-the-logit assumption for continuous predictors was evaluated using Box–Tidwell-type interaction terms. Model fit was evaluated using the akaike information criterion. All statistical tests were 2-sided, and *P* values were reported to 3 decimal places, with values <.001 reported as such. All analyses were performed using R software (R Foundation for Statistical Computing, version 4.5.2).^[24]^

## 3. Results

### 3.1. Baseline characteristics

A total of 138 patients with T2DM were included in the analytical cohort. Of these, 41 (29.7%) had HbA1c ≤7% and 97 (70.3%) had HbA1c >7%. The mean age was 67.3 ± 11.5 years, 99 patients (71.7%) were male, and 128 (92.8%) were affiliated with the subsidized healthcare system (Table 1). Patients with HbA1c >7% were older than those with HbA1c ≤7% (68.6 ± 11.6 vs 64.2 ± 10.9 years; *P* = .034) (Fig. 2).

**Table 1:** Baseline sociodemographic and clinical characteristics according to HbA1c group.

Characteristic	Overall n = 138*	HbA1c ≤ 7% n = 41*	HbA1c > 7% n = 97*	*P* value
Age, yr	67.3 ± 11.5	64.2 ± 10.9	68.6 ± 11.6	.034†
Duration of T2DM, yr	4.6 ± 5.0	5.7 ± 5.2	4.5 ± 5.0	.461†
Missing	59	30	29	
Sex				.068‡
Female	39 (28.3%)	16 (39.0%)	23 (23.7%)	
Male	99 (71.7%)	25 (61.0%)	74 (76.3%)	
Health insurance regime				.281§
Contributory	10 (7.2%)	1 (2.4%)	9 (9.3%)	
Subsidized	128 (92.8%)	40 (97.6%)	88 (90.7%)	
Hypertension	54 (93.1%)	27 (90.0%)	27 (96.4%)	.612§
Missing	80	11	69	
Ischemic heart disease	64 (46.4%)	24 (58.5%)	40 (41.2%)	.063‡
Heart failure	52 (37.7%)	18 (43.9%)	34 (35.1%)	.327‡
Clinical atrial fibrillation	12 (8.7%)	5 (12.2%)	7 (7.2%)	.340§
Cerebrovascular disease	11 (8.0%)	0 (0.0%)	11 (11.3%)	.034§

HbA1c = glycated hemoglobin, T2DM = type 2 diabetes mellitus.

* Mean ± SD; n (%).

† Welch 2 sample *t* test.

‡ Pearson’s chi-squared test.

§ Fisher’s exact test.

**Figure 2.**
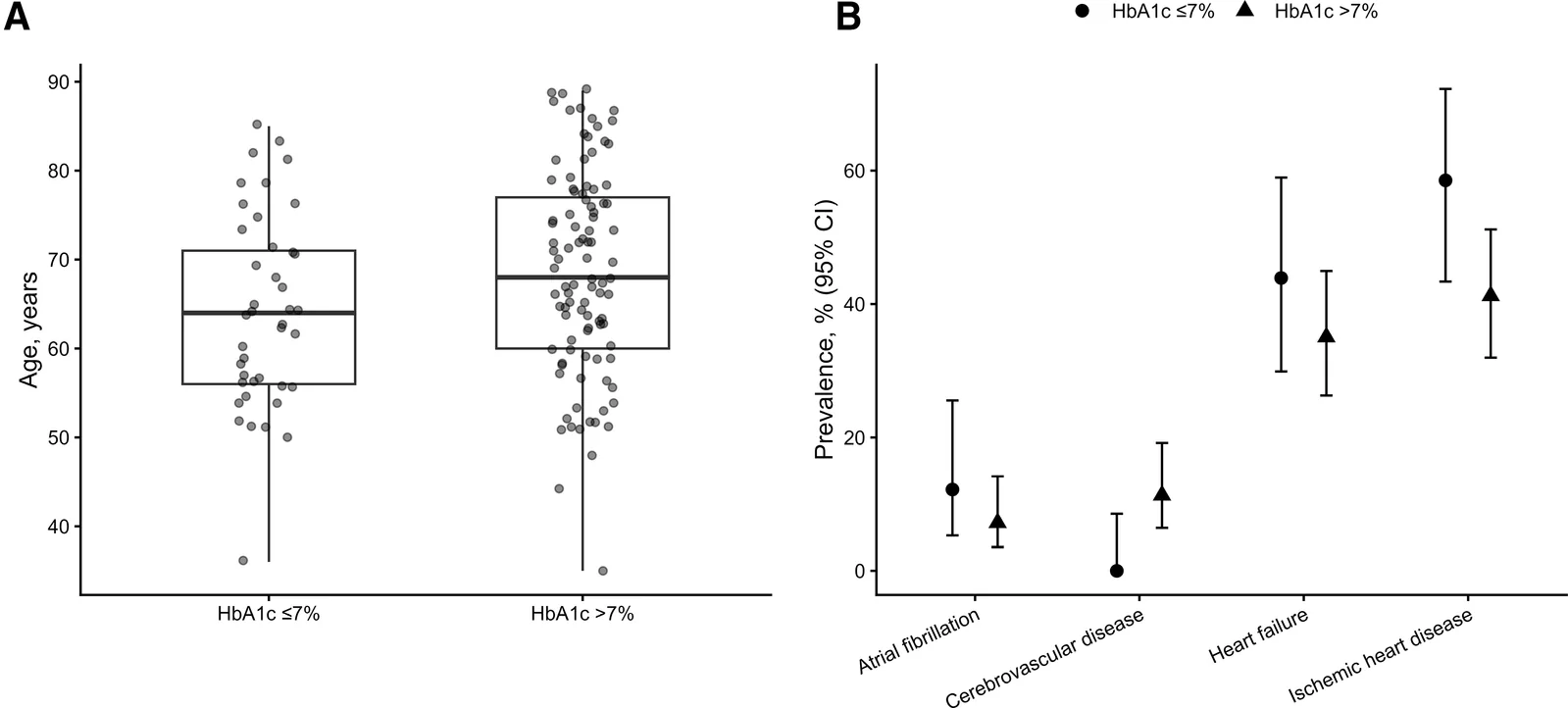
(A) Age and (B) comorbidities according to HbA1c group. CI = confidence interval, HbA1c = glycated hemoglobin.

Cerebrovascular disease was observed in 11.3% of patients with HbA1c >7% and in none of those with HbA1c ≤7% (*P* = .034). IHD was present in 41.2% and 58.5% of patients, respectively (*P* = .063), while no statistically significant between-group differences were observed for HF or clinical AF. Hypertension was frequent among patients with available data, although this variable had substantial missingness.

### 3.2. Laboratory evaluation

The overall mean HbA1c was 8.4 ± 1.9%; as HbA1c defined the analytical groups, no inferential *P* value was calculated for its between-group comparison (Table 2). Patients with HbA1c >7% had higher mean total cholesterol (203.5 ± 48.1 vs 181.7 ± 42.8 mg/dL; *P* = .010) and low-density lipoprotein cholesterol (124.9 ± 36.1 vs 106.0 ± 39.1 mg/dL; *P* = .010) than those with HbA1c ≤7%.

**Table 2:** Glycated hemoglobin, lipid profile, and renal function according to HbA1c group.

Characteristic	Overall n = 138*	HbA1c ≤ 7% n = 41*	HbA1c > 7% n = 97*	*P* value
HbA1c, % (descriptive only)	8.4 ± 1.9	6.3 ± 0.5	9.3 ± 1.5	
Total cholesterol, mg/dL	197.0 ± 47.5	181.7 ± 42.8	203.5 ± 48.1	.010†
LDL cholesterol, mg/dL	119.3 ± 37.9	106.0 ± 39.1	124.9 ± 36.1	.010†
HDL cholesterol, mg/dL	47.6 ± 12.6	49.6 ± 11.5	46.7 ± 12.9	.193†
Triglycerides, mg/dL	184.9 ± 78.1	171.7 ± 95.8	190.4 ± 69.1	.261†
Creatinine, mg/dL	1.0 ± 0.4	1.1 ± 0.4	1.0 ± 0.4	.562†
eGFR (MDRD-4), mL/min/1.73 m^2^	75.4 ± 27.5	72.0 ± 27.6	76.9 ± 27.4	.351†

eGFR = estimated glomerular filtration rate, HbA1c = glycated hemoglobin, HDL = high-density lipoprotein, LDL = low-density lipoprotein.

* Mean ± SD.

† Welch 2 sample *t* test.

No statistically significant differences were observed for high-density lipoprotein cholesterol (*P* = .193), triglycerides (*P* = .261), serum creatinine (*P* = .562), or estimated glomerular filtration rate (*P* = .351).

### 3.3. ECG and TTE

Table 3 summarizes the electrocardiographic and TTE findings. Sinus rhythm was documented in 62 patients (44.9%), without a significant difference between the HbA1c ≤7% and >7% groups (36.6% vs 48.5%; *P* = .200). No significant between-group differences were observed for conduction abnormalities, supraventricular extrasystoles, electrocardiographic left ventricular hypertrophy, or AF on the index ECG.

**Table 3:** Electrocardiographic and transthoracic echocardiographic findings according to HbA1c group.

Characteristic	Overall n = 138*	HbA1c ≤ 7% n = 41*	HbA1c > 7% n = 97*	*P* value
Sinus rhythm	62 (44.9%)	15 (36.6%)	47 (48.5%)	.200†
Conduction abnormalities				.853‡
None	114 (82.6%)	36 (87.8%)	78 (80.4%)	
First-degree AV block	6 (4.3%)	1 (2.4%)	5 (5.2%)	
Right bundle branch block	4 (2.9%)	1 (2.4%)	3 (3.1%)	
Left bundle branch block	14 (10.1%)	3 (7.3%)	11 (11.3%)	
Supraventricular extrasystoles	8 (5.8%)	1 (2.4%)	7 (7.2%)	.436‡
Left ventricular hypertrophy (ECG)	8 (5.8%)	4 (9.8%)	4 (4.1%)	.237‡
Atrial fibrillation on index ECG	2 (1.4%)	1 (2.4%)	1 (1.0%)	.507‡
LVEF, %	52.1 ± 12.7	51.5 ± 12.5	52.4 ± 12.8	.724§
LVEF 40%–49%	14 (10.1%)	6 (14.6%)	8 (8.2%)	.354‡
LVEF < 40%	32 (23.2%)	9 (22.0%)	23 (23.7%)	.823†
Absence of prespecified echocardiographic abnormalities	29 (21.0%)	14 (34.1%)	15 (15.5%)	.014†
Hypokinesia	26 (18.8%)	9 (22.0%)	17 (17.5%)	.543†
Left ventricular hypertrophy (echocardiography)	30 (21.7%)	6 (14.6%)	24 (24.7%)	.188†
Diastolic dysfunction	37 (26.8%)	8 (19.5%)	29 (29.9%)	.208†
Systolic dysfunction	22 (15.9%)	4 (9.8%)	18 (18.6%)	.197†

AV = atrioventricular, HbA1c = glycated hemoglobin, ECG = electrocardiogram, LVEF = left ventricular ejection fraction.

* n (%); mean ± SD.

† Pearson’s chi-squared test.

‡ Fisher’s exact test.

§ Welch 2 sample *t* test.

Clinical AF documented in the medical history was present in 12 patients (8.7%) (Table 1), whereas AF was present on the index ECG in only 2 patients (1.4%) (Table 3). These variables represent different assessments: the former reflects a documented clinical diagnosis, whereas the latter reflects the rhythm recorded at the index electrocardiographic examination.

The absence of prespecified echocardiographic abnormalities was less frequent among patients with HbA1c > 7% than among those with HbA1c ≤ 7% (15.5% vs 34.1%; *P* = .014) (Fig. 3). Mean LVEF was similar between groups (52.4 ± 12.8% vs 51.5 ± 12.5%; *P* = .724). Systolic dysfunction was observed in 18.6% versus 9.8% (*P* = .197), and diastolic dysfunction in 29.9% versus 19.5% (*P* = .208), respectively; neither difference reached statistical significance.

**Figure 3.**
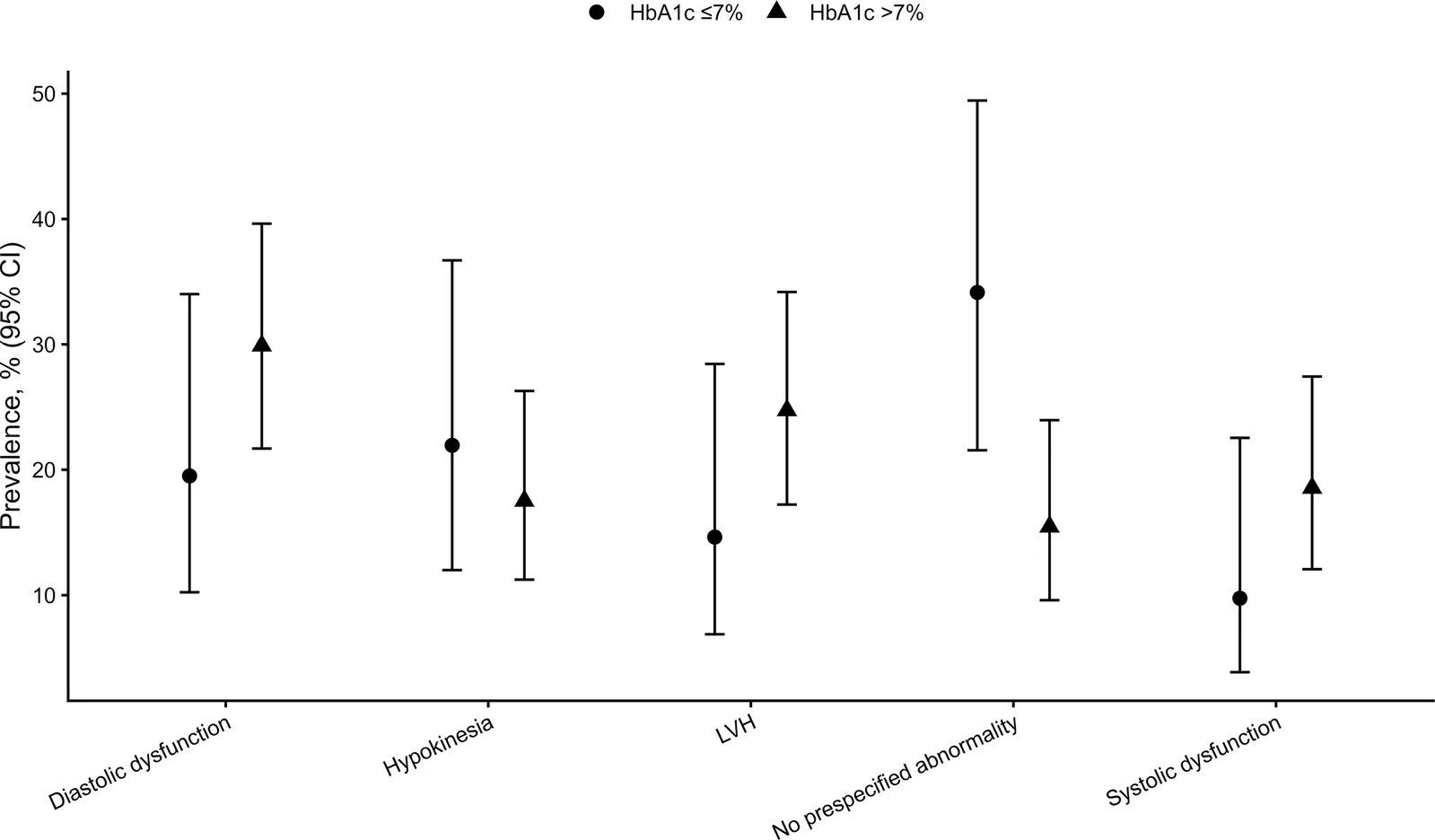
Echocardiographic findings according to HbA1c group. CI = confidence interval, HbA1c = glycated hemoglobin, LVH = left ventricular hypertrophy.

### 3.4. Pharmacological treatment

Documented prescribed medication use was high for statins (96.4%), ACE inhibitors or ARB (89.9%), beta-blockers (82.6%), and antiplatelet agents (66.7%), with no statistically significant differences between HbA1c groups (Table 4).

**Table 4:** Documented pharmacological treatment according to HbA1c group.

Documented medication use	Overall n = 138*	HbA1c ≤ 7% n = 41*	HbA1c > 7% n = 97*	*P* value
ACE inhibitor/ARB	124 (89.9%)	35 (85.4%)	89 (91.8%)	.354†
Beta-blocker	114 (82.6%)	37 (90.2%)	77 (79.4%)	.124‡
Statin	133 (96.4%)	38 (92.7%)	95 (97.9%)	.155†
Antiplatelet agent	92 (66.7%)	26 (63.4%)	66 (68.0%)	.598‡
Amiodarone	1 (0.7%)	1 (2.4%)	0 (0.0%)	.297†
Direct oral anticoagulant (DOAC)	1 (0.7%)	0 (0.0%)	1 (1.0%)	1.000†
SGLT2 inhibitor	23 (16.7%)	11 (26.8%)	12 (12.4%)	.037‡

ACE = angiotensin-converting enzyme, ARB = angiotensin receptor blocker, HbA1c = glycated hemoglobin, SGLT2 = sodium–glucose cotransporter-2.

* n (%); mean ± SD.

† Fisher’s exact test.

‡ Pearson’s chi-squared test.

SGLT2 inhibitor use was documented in 23 patients (16.7%) and was more frequent among patients with HbA1c ≤7% than among those with HbA1c >7% (26.8% vs 12.4%; *P* = .037) (Fig. 4). Amiodarone and direct oral anticoagulant use were uncommon, each being documented in 1 patient (0.7%).

**Figure 4.**
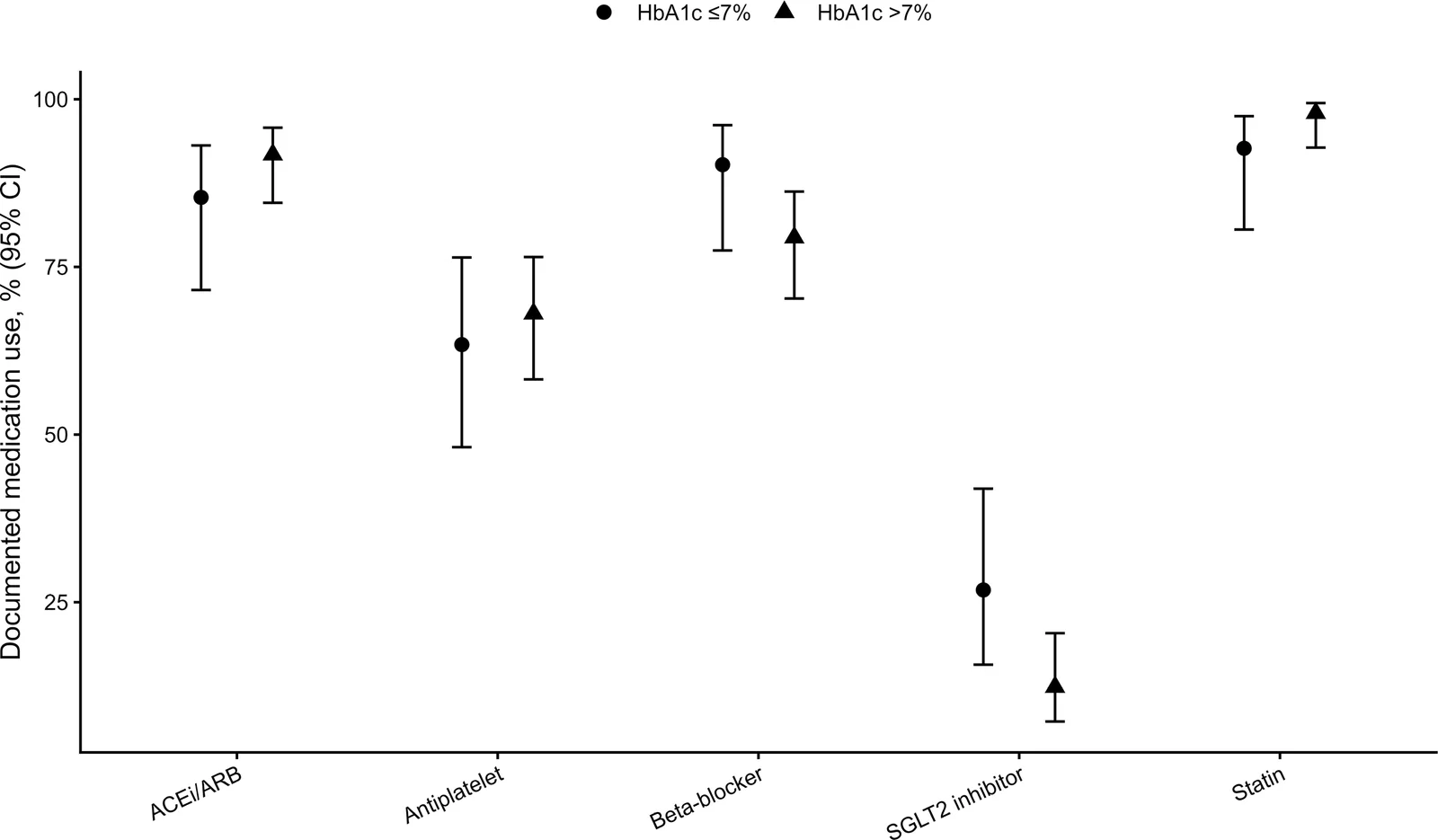
Documented medication use according to HbA1c group. ACEi = angiotensin-converting enzyme inhibitor, ARB = angiotensin receptor blocker, CI = confidence interval, HbA1c = glycated hemoglobin, SGLT2 = sodium–glucose cotransporter 2.

### 3.5. Factors associated with HbA1c >7%

Univariable and multivariable logistic regression analyses are presented together in Table 5. In univariable analyses, age was associated with higher odds of HbA1c >7% per 10-year increment (OR = 1.41; 95% CI: 1.01–1.97; *P* = .041), as was total cholesterol per 10-mg/dL increment (OR = 1.11; 95% CI: 1.02–1.21; *P* = .015). Absence of prespecified echocardiographic abnormalities (OR = 0.35; 95% CI: 0.15–0.82; *P* = .016) and SGLT2 inhibitor use (OR = 0.39; 95% CI: 0.15–0.96; *P* = .042) were inversely associated with HbA1c >7%. For CVD, the conventional logistic OR was not estimable because of complete separation; Fisher’s exact test yielded *P* = .034.

**Table 5:** Univariable and multivariable logistic regression analyses for factors associated with HbA1c > 7%.

Predictor	Univariable analysis			Multivariable analysis		
	OR	95% CI	*P* value	aOR	95% CI	*P* value
Age, per 10 yr	1.41	1.01–1.97	.041	1.35	0.93–1.94	.113
Male sex	2.06	0.94–4.50	.071	1.77	0.75–4.17	.194
Subsidized insurance	0.24	0.03–1.99	.188	–	–	–
Ischemic heart disease	0.50	0.24–1.04	.065	–	–	–
Heart failure	0.69	0.33–1.45	.328	–	–	–
Cerebrovascular disease	NE	–	.034*	–	–	–
Total cholesterol, per 10 mg/dL	1.11	1.02–1.21	.015	1.10	1.01–1.20	.033
Triglycerides, per 50 mg/dL	1.18	0.92–1.52	.199	–	–	–
LVEF < 40%	1.11	0.46–2.65	.823	–	–	–
Absence of prespecified echocardiographic abnormalities	0.35	0.15–0.82	.016	0.36	0.15–0.88	.026
ACEi/ARB use	1.91	0.62–5.89	.262	–	–	–
Beta-blocker use	0.42	0.13–1.31	.133	–	–	–
Statin use	3.75	0.60–23.34	.157	–	–	–
SGLT2 inhibitor use	0.39	0.15–0.96	.042	0.56	0.20–1.58	.274

Adjusted estimates are reported only for variables included in the prespecified exploratory multivariable model: age, sex, total cholesterol, absence of prespecified echocardiographic abnormalities, and SGLT2 inhibitor use. All variables were entered simultaneously using the enter method.

ACEi = angiotensin-converting enzyme inhibitor, aOR = adjusted odds ratio, ARB = angiotensin receptor blocker, CI = confidence interval, HbA1c = glycated hemoglobin, LVEF = left ventricular ejection fraction, NE = not estimable, OR = odds ratio, SGLT2 = sodium–glucose cotransporter 2.

* *P*-value calculated using Fisher’s exact test; the conventional logistic regression OR was not estimable because of complete separation.

In the exploratory multivariable model, total cholesterol remained associated with HbA1c >7%, with each 10-mg/dL increase corresponding to an adjusted OR of 1.10 (95% CI: 1.01–1.20; *P* = .033). Absence of prespecified echocardiographic abnormalities was inversely associated with HbA1c >7% (adjusted OR = 0.36; 95% CI: 0.15–0.88; *P* = .026). Age (adjusted OR = 1.35; 95% CI: 0.93–1.94; *P* = .113), male sex (adjusted OR = 1.77; 95% CI: 0.75–4.17; *P* = .194), and SGLT2 inhibitor use (adjusted OR = 0.56; 95% CI: 0.20–1.58; *P* = .274) were not statistically significant after adjustment.

Results were similar in the sensitivity analysis that additionally included IHD (Table S1, Supplemental Digital Content 1). Total cholesterol remained associated with higher odds of HbA1c >7% (adjusted OR = 1.10; 95% CI: 1.01–1.20; *P* = .035), whereas absence of prespecified echocardiographic abnormalities remained inversely associated with the outcome (adjusted OR = 0.37; 95% CI: 0.15–0.93; *P* = .034). IHD was not significantly associated with HbA1c >7% (adjusted OR = 0.70; 95% CI: 0.31–1.59; *P* = .396).

## 4. Discussion

This study provides real-world evidence on the cardiovascular profile and treatment patterns of patients with T2DM receiving specialized outpatient cardiology care in Barranquilla, Colombia. Most participants were older men affiliated with the subsidized healthcare system, and 70.3% had HbA1c >7%. In univariable analyses, older age and higher total cholesterol were associated with HbA1c >7%, whereas CVD occurred exclusively in this group. In the exploratory multivariable model, total cholesterol remained positively associated with HbA1c >7%, while the absence of prespecified echocardiographic abnormalities was inversely associated with this outcome. These findings are consistent with evidence emphasizing the influence of social determinants on diabetes outcomes,^[25,26]^ while Colombian and international real-world studies have documented the relevance of multimorbidity and cardiovascular treatment complexity in patients with T2DM.^[9,27]^

The association between higher total cholesterol and HbA1c >7% is consistent with the clustering of metabolic abnormalities commonly observed in T2DM and with the established relationship between glycemia, dyslipidemia, and cardiovascular risk.^[4,5,9]^ Age was associated with HbA1c >7% in the univariable analysis but not after multivariable adjustment, suggesting that its relationship with glycemic status may partly reflect the broader clinical complexity of older patients. Likewise, CVD was observed only among patients with HbA1c >7%; however, the resulting complete separation precluded conventional logistic estimation. Previous longitudinal and meta-analytic evidence has linked increasing glycemic exposure with macrovascular complications and stroke risk.^[4,10,28]^ Nevertheless, the present findings should be interpreted as cross-sectional associations rather than evidence that glycemic status caused the observed cardiovascular conditions.

Echocardiographic findings also differed according to HbA1c group. The absence of prespecified echocardiographic abnormalities was less frequent among patients with HbA1c >7% and remained inversely associated with this outcome after adjustment. This observation is consistent with previous evidence describing structural and functional myocardial abnormalities in T2DM, including diabetic cardiomyopathy, diastolic dysfunction, and HF phenotypes.^[6,7,29–31]^ Studies specifically examining HbA1c have also reported associations between higher glycemic levels and left ventricular diastolic dysfunction.^[30]^ Nevertheless, the composite used in this study should not be interpreted as a completely normal echocardiogram or as a protective factor. Because the study was cross-sectional, neither the direction nor the temporality of the association between glycemic status and cardiac abnormalities can be established.^[32]^

Documented prescribed use of conventional cardiovascular therapies was high, particularly for statins, ACE inhibitors/ARBs, and beta-blockers, broadly consistent with contemporary cardiovascular risk-management recommendations.^[12,14]^ In contrast, SGLT2 inhibitors were prescribed to only 16.7% of the cohort and were more frequently documented among patients with HbA1c ≤7%. Relatively low use of SGLT2 inhibitors and other glucose-lowering therapies with cardiovascular benefit has also been described in international real-world studies, including DISCOVER and large healthcare-system cohorts.^[16–18,27]^ Moreover, socioeconomic differences in SGLT2 inhibitor utilization have been reported, supporting the potential relevance of access-related factors in vulnerable populations.^[19]^

Although SGLT2 inhibitor use was inversely associated with HbA1c >7% in the univariable analysis, this association was attenuated and was no longer statistically significant after multivariable adjustment (adjusted OR = 0.56; 95% CI: 0.20–1.58). Accordingly, this finding should not be interpreted as evidence of a treatment effect. Current American Diabetes Association and European Society of Cardiology recommendations support the use of therapies with demonstrated cardiovascular and cardiorenal benefits according to the patient’s clinical profile, rather than solely on the basis of glycemic status.^[12–14]^ From a clinical perspective, these real-world findings support systematic assessment of lipid management, cardiovascular comorbidities, and eligibility for evidence-based cardiometabolic therapies during specialized cardiology follow-up.

Several limitations should be considered. First, the cross-sectional design precludes assessment of temporality or causality.^[32]^ Second, the modest sample size limited statistical power and precision, and the multivariable model should therefore be considered exploratory and hypothesis-generating. This limitation is particularly relevant for SGLT2 inhibitors, which were documented in only 23 patients, resulting in a relatively wide CI for the adjusted estimate and limited ability to detect an independent association. Third, selection bias is possible because 177 of the 315 patients with confirmed T2DM were excluded because of incomplete medical records. The analytical cohort may therefore disproportionately represent patients with more complete follow-up or more extensive cardiovascular evaluation. Fourth, the single-center setting and predominance of patients from the subsidized healthcare system may limit generalizability. In addition, the retrospective records did not systematically capture potentially relevant factors such as body mass index, complete information on diabetes duration, actual medication adherence, and detailed severity of comorbidities. Finally, the HbA1c threshold of 7% was used as a standardized analytical cutoff and should not be interpreted as an individualized therapeutic target, particularly in older or multimorbid patients, for whom contemporary guidelines recommend individualized glycemic goals.^[21]^

Despite these limitations, this study contributes real-world cardiovascular data from an underrepresented Colombian population and integrates clinical, laboratory, electrocardiographic, echocardiographic, and treatment information within a specialized outpatient setting. Previous Colombian studies have examined glycemic control, cardiovascular events, and clinical inertia,^[9,10,15]^ but evidence integrating these domains within specialized cardiology care remains limited. More broadly, the relative underrepresentation of Latin American cardiovascular research reinforces the value of locally generated evidence.^[33]^ Prospective multicenter studies with larger samples, more complete assessment of treatment exposure and adherence, and longitudinal cardiovascular outcomes are needed to determine the clinical significance and temporal relationships of these observations.

## 5. Conclusions

In this single-center real-world cohort of Colombian patients with T2DM receiving specialized cardiology outpatient care, 70.3% had HbA1c >7%, within a population characterized by a substantial cardiovascular comorbidity burden. Higher total cholesterol was independently associated with HbA1c >7%, whereas the absence of prespecified echocardiographic abnormalities was inversely associated with this outcome. These findings represent cross-sectional associations and should not be interpreted as evidence of temporality, causality, or a protective effect. Documented SGLT2 inhibitor use was limited and was more frequent among patients with HbA1c ≤7%; however, this association was no longer statistically significant after multivariable adjustment and therefore should not be interpreted as a treatment effect. These real-world findings support integrated cardiometabolic assessment and optimization of evidence-based treatment in specialized outpatient care, while larger prospective multicenter studies are needed to confirm these associations.

## Acknowledgments

The authors acknowledge the support of Clínica Centro and Universidad Simón Bolívar in the development of this study. We also thank the Internal Medicine Residency Program for providing the academic and clinical environment that enabled this research.

## Author contributions

**Conceptualization:** Lucia Suarez Maestre, Ingrith Paola Hoyos Montaño, Zenen David Rúa Osorio, Alex Dominguez Vargas, Henry J. González-Torres.

**Data curation:** Lucia Suarez Maestre, Ingrith Paola Hoyos Montaño, Zenen David Rúa Osorio, Henry J. González-Torres.

**Formal analysis:** Zenen David Rúa Osorio, Alex Dominguez Vargas, Henry J. González-Torres.

**Investigation:** Lucia Suarez Maestre, Ingrith Paola Hoyos Montaño.

**Methodology:** Lucia Suarez Maestre, Ingrith Paola Hoyos Montaño, Alex Dominguez Vargas, Henry J. González-Torres.

**Project administration:** Carlos Renowitzky Zabarain.

**Resources:** Carlos Renowitzky Zabarain.

**Supervision:** Zenen David Rúa Osorio, Carlos Renowitzky Zabarain, Henry J. González-Torres.

**Writing – original draft:** Lucia Suarez Maestre, Ingrith Paola Hoyos Montaño, Zenen David Rúa Osorio, Carlos Renowitzky Zabarain, Alex Dominguez Vargas, Henry J. González-Torres.

**Writing – review & editing:** Lucia Suarez Maestre, Ingrith Paola Hoyos Montaño, Zenen David Rúa Osorio, Carlos Renowitzky Zabarain, Alex Dominguez Vargas, Henry J. González-Torres.


