# Supplementary material for: Cardiovascular risk profile and treatment patterns in patients with type 2 diabetes in specialized cardiology outpatient care in Colombia: A retrospective cross-sectional study
Source: Medicine (Baltimore). 2026 Oct 23;105(43):e51307. doi: 10.1097/MD.0000000000051307 (PMC13706202; doi:10.1097/MD.0000000000051307)
Supplement: Supplementary file 1 [file medi-105-e51307-s001.docx]

**Supplementary Table S1.** Sensitivity analysis of the exploratory multivariable logistic regression model additionally including ischemic heart disease.

| **Predictor** | **Adjusted OR** | **95% CI** | **p-value** |
| --- | --- | --- | --- |
| Age, per 10 years | 1.35 | 0.93–1.95 | 0.113 |
| Male sex | 1.67 | 0.70–3.99 | 0.247 |
| Ischemic heart disease | 0.70 | 0.31–1.59 | 0.396 |
| Total cholesterol, per 10 mg/dL | 1.10 | 1.01–1.20 | 0.035 |
| Absence of prespecified echocardiographic abnormalities | 0.37 | 0.15–0.93 | 0.034 |
| SGLT2 inhibitor use | 0.61 | 0.22–1.73 | 0.356 |

**Note:** The sensitivity model additionally included ischemic heart disease together with age, sex, total cholesterol, absence of prespecified echocardiographic abnormalities, and SGLT2 inhibitor use. All variables were entered simultaneously using the enter method. The dependent outcome was HbA1c >7%.

**Abbreviations:** CI, confidence interval; HbA1c, glycated hemoglobin; OR, odds ratio; SGLT2, sodium–glucose cotransporter 2.
